# NLRP3 Inflammasome-Mediated Pyroptosis Pathway Contributes to the Pathogenesis of *Candida albicans* Keratitis

**DOI:** 10.3389/fmed.2022.845129

**Published:** 2022-04-06

**Authors:** Huifang Lian, XiaoLong Fang, Qingyu Li, Shuang Liu, Qiuhong Wei, Xia Hua, Wenguang Li, Chunyang Liao, Xiaoyong Yuan

**Affiliations:** ^1^Clinical College of Ophthalmology, Tianjin Medical University, Tianjin, China; ^2^Tianjin Eye Hospital, Tianjin Eye Institute, Tianjin Key Laboratory of Ophthalmology and Visual Science, Tianjin, China; ^3^Department of Ophthalmology, Baoding First Central Hospital, Baoding, China; ^4^State Key Laboratory of Environmental Chemistry and Ecotoxicology, Research Center for Eco-Environmental Sciences, Chinese Academy of Sciences, Beijing, China; ^5^The School of Medicine, Nankai University, Tianjin, China; ^6^University of Chinese Academy of Sciences, Beijing, China; ^7^Aier Eye Hospital, Tianjin, China; ^8^Institute of Genetics and Developmental Biology, Chinese Academy of Sciences, Beijing, China

**Keywords:** NLRP3 inflammasome, pyroptosis, *Candida albicans*, keratitis, corneal epithelial cells

## Abstract

**Purpose:**

Fungal keratitis is a sight-threatening corneal infection caused by fungal pathogens, and the pathogenic mechanisms have not been fully elucidated. The aim of this study was to determine whether NOD-like receptor family pyrin domain containing 3 (NLRP3) inflammasome-mediated pyroptosis contributes to *Candida albicans* (*C. albicans*) keratitis and explore the underlying mechanism.

**Methods:**

An *in vivo* mouse model of *C. albicans* keratitis and an *in vitro* culture model of human corneal epithelial cells (HCECs) challenged with heat-killed *C. albicans* (HKCA) were established in this study. The degree of corneal infection was evaluated by clinical scoring. Gene expression was assessed using reverse transcription-quantitative polymerase chain reaction (RT-qPCR) and western blot analysis or immunofluorescence staining was performed to evaluate protein expression. TdT-mediated dUTP nick end labeling (TUNEL) staining was performed to examine the pyroptotic cell death. A lactate dehydrogenase (LDH) release assay was performed to assess cytotoxicity.

**Results:**

Compared with the mock-infected group, we observed that the mRNA levels of NLRP3, caspase-1 (CASP1), interleukin (IL)−1β and gasdermin-D (GSDMD) in *C. albicans*-infected mice cornea was significantly increased. Our data also demonstrated that the protein expression of NLRP3 and the pyroptosis-related markers apoptosis-associated speck-like protein containing a CARD (ASC), cleaved CASP1, N-GSDMD, cleaved IL-1β and cleaved IL-18 as well as pyroptotic cell death were dramatically elevated in the mouse model of *C. albicans* keratitis. More importantly, NLRP3 knockdown markedly alleviated pyroptosis and consequently reduced corneal inflammatory reaction in *C. albicans* keratitis. *In vitro*, the presence of activated NLRP3 inflammasome and pyroptotic cell death were validated in HCECs exposed to HKCA. Furthermore, the potassium (K^+^) channel inhibitor glyburide decreased LDH release and suppressed NLRP3 inflammasome activation and pyroptosis in HCECs exposed to HKCA.

**Conclusion:**

In conclusion, the current study revealed for the first time that NLRP3 inflammasome activation and pyroptosis occur in *C. albicans*-infected mouse corneas and HCECs. Moreover, NLRP3 inflammasome-mediated pyroptosis signaling is involved in the disease severity of *C. albicans* keratitis. Therefore, This NLRP3 inflammasome-dependent pathway may be an attractive target for the treatment of fungal keratitis.

## Introduction

Fungal keratitis is a corneal infection disease caused by pathogenic fungi that is often accompanied by moderate or worsened visual impairment and even blindness (1, 2). Recently, with the wide use of contact lenses and the abuse of glucocorticoids and antibiotics, *Candida albicans* (*C. albicans*) keratitis has become a worldwide problem, and its incidence is on the rise (3). Compared with that of other filamentous fungal infections, the clinical presentation of keratitis caused by *C. albicans* infection is often more striking because it usually occurs in patients with preexisting corneal disorders (4, 5). Thus, it is important to fully understand the pathogenesis of fungal keratitis and generate novel treatments to fight this disease.

Innate immunity is the frontline of defense in the cornea against fungal infection. As the main component of the corneal innate immune system, corneal epithelial cells are able to sense and respond to fungal stimuli and participate in the recognition of pathogen-associated molecular patterns (PAMPs) or damage-associated molecular patterns (DAMPs) through pattern recognition receptors (PRRs), leading to a robust inflammatory reaction (6). NOD-like receptor family pyrin domain containing 3 (NLRP3), an intracellular PRR, has been shown to participate in inflammasome formation with an adaptor protein (apoptosis-associated speck like protein containing a caspase recruitment domain, ASC) and an effector pro-caspase-1 (pro-CASP1) and is primarily expressed in immune cells (7, 8). Upon activation, the NLRP3 inflammasome leads to the cleavage of CASP1, which processes pro-IL-1β and pro-IL-18 to their mature forms and cleaves gasdermin-D (GSDMD) into a pore-forming amino-terminal domain (N-GSDMD) form triggering pyroptosis (9). Pyroptosis is an inflammasome-mediated programmed cell death that is characterized by pyroptotic cell lysis and the release of proinflammatory cytokines (10) and is critical in the pathological mechanism of *C. albicans* infection (9, 11).

Currently, studies have shown that activation of the NLRP3 inflammasome plays a crucial role in the innate immune response to *C. albicans* infection (12, 13). Furthermore, activation of the NLRP3 inflammasome is required for *C. albicans*-induced pyroptosis in myeloid or epithelial cells (11, 14). In recent years, the NLRP3 inflammasome has been implicated as an innate sensor that is expressed on the corneal surface and contributes to the pathogenesis of various ocular disorders, such as *Aspergillus fumigatus* (*A. fumigatus*) and *Fusarium* keratitis, dry eye and corneal alkali burn (15–19). Moreover, the pyroptosis effector GSDMD has been reported to be involved in corneal epithelial cell responses to *A. fumigatus* and hyperosmotic stress (19, 20). However, the precise role of the NLRP3 inflammasome in corneal epithelial cells in response to *C. albicans* has not been elucidated so far. Moreover, whether the NLRP3 inflammasome affects pyroptosis in fungal keratitis remains uncertain.

Therefore, the goal of this study was to explore whether NLRP3 inflammasome activation and pyroptosis are present in *C. albicans*-infected mouse corneas and human corneal epithelial cells (HCECs) and contribute to the pathogenesis of *C. albicans* keratitis and the potential mechanism.

## Materials and Methods

### Fungi

*C. albicans* strain SC5314 was purchased from China General Microbiological Culture Collection Center (Beijing, China) and cultured on YPD agar for 3 days at 25°C. Colonies were collected after 3 days of culture and diluted in sterile phosphate-buffered saline (PBS) to yield 1 × 10^6^ colony-forming units (CFU)/5 μL according to the optical density (OD), which was measured at 600 nm, and a predetermined OD600 conversion factor of 1 OD = 3 × 10^7^ CFU/mL (21). For heat inactivation, *C. albicans* was resuspended in PBS and incubated at 95°C for 30 min. Heat-killed *C. albicans* (HKCA) cells were diluted to different concentrations with DMEM prior to be administered to HCECs.

### Animals

Wild-type C57BL/6N mice (female, 6–8 weeks of age) were purchased from Vital River Laboratory Animal Technology Co., Ltd. (Beijing, China). The mice were anesthetized with 50 mg/kg pentobarbital via intraperitoneal injection. The right corneas were scratched by a 22-gauge needle, and then 5 μL of 1 × 10^6^ CFU of *C. albicans* was inoculated onto the scratched cornea to induce keratitis (22). Animals that served as mock-infected controls were inoculated with sterile PBS. The mice were monitored daily for 1 week post-inoculation (p.i.) using slit lamp to evaluate corneal infection and the clinical scores (ranging from 0 to 12); a grading scale of 0 to 4 was assigned to each of the following three aspects: area of opacity, opacity density, and surface regularity of the corneas. A total score of 0–5 signaled mild eye disease, 6–9 indicated moderate disease, and 10–12 represented severe disease (23). *C. albicans* keratitis was created at day 0 and mice were sacrificed at 1, 3, and 7 days post-inoculation (dpi), and the right eyes were enucleated for analysis. Animal treatments were conducted strictly in accordance with the ARVO Statement for the Use of Animals in Ophthalmic and Vision Research. All experimental protocols related to animals were approved by the Research Center for Eco-Environmental Sciences, Chinese Academy of Sciences.

### NLRP3 Knockdown Experiments

The green fluorescent protein (GFP)-tagged pAdTrack adenovirus carrying negative small hairpin RNA (Ad-GFP-shRNA, served as a control) or NLRP3 shRNA (Ad-NLRP3-shRNA) was constructed by Quanyang Biotechnology Co., Ltd. (Shanghai, China). The sequences of the NLRP3 shRNA were designed according to GenBank accession number NM_145827.4, and the adenovirus particles contained a pool of three different NLRP3 shRNA sequences (1, 2 and 3). The sequence information is as follows:

mNLRP3-shRNA-1: 5'- CCAGGAGAGAACCTCTTATTT-3';

mNLRP3-shRNA-2: 5'- GGATCTTTGCTGCGATCAACA-3'; and

mNLRP3-shRNA-3: 5'- GCGAGAGATTCTACAGCTTCA-3'.

The experimental eye of each mouse was injected subconjunctivally with 6 μL (4 × 10^8^ PFU) of the Ad-GFP-shRNA or Ad-NLRP3-shRNA suspension 3 days prior to *C. albicans* infection. On day 3 p.i., the mice were euthanized, and their corneas and eyeballs were harvested for further experiments.

### HCEC Culture and Treatment

HCECs were obtained from ATCC (Manassas, VA, USA). The HCECs were cultured in DMEM (Gibco, #C11995500BT) supplemented with 10% FBS (Gibco, #10099141), 1% penicillin–streptomycin (Gibco, #15140122), 10 ng/mL hEGF (Sino Biological Inc., #10605-H01H), 5 μg/mL transferrin (Sigma–Aldrich, #T2036) and 5 μg/mL insulin (Solarbio, #I8830) at 37°C in 5% CO_2_. The HCECs were then seeded onto 6-well at a density of 1 × 10^6^ cells/well. When the cells reached 80-90% confluence, the cell culture medium was switched to serum-free DMEM, after which the HECEs were incubated overnight and then treated for 0 (control), 2, 4, 8, 12, 24, or 36 h with multiple concentrations HKCA (10^3^-10^7^ CFU/mL), equivalent to a multiplicity of infection (MOI, the ratio of yeast cells to corneal epithelial cells) of 1:500, 1:50, 1:5, 2:1, or 20:1 respectively. In the potassium (K^+^) channel blocking experiments, HCECs were seeded in 6-well plates at a density of 1 × 10^6^ cells/well and pretreated with glyburide (Sigma–Aldrich, #G0639) for 2 h and then stimulated with HKCA (MOI = 20) for 24 h. Untreated cells that were incubated with 0.1% DMSO (MP Biomedicals, #196055) were used as the control group.

### Lactate Dehydrogenase (LDH) Release Assay

The increased release of LDH was used as an indicator of the loss of cell membrane integrity. HCECs were seeded in 96-well plates at a density of 5 × 10^4^ cells/well and infected with HKCA (MOI = 20) for 24h. The cell supernatants were collected at the specified time points, and LDH release was assessed using an LDH release assay kit (Nanjing Jiancheng Bioengineering Institute, Nanjing, China) according to the manufacturer's instructions. The absorbance of the samples was measured at 450 nm using a multifunctional microplate reader (Thermo Scientific, USA). The results are expressed as a percentage of the maximum LDH release, obtained by lysing the cells in 1% Triton X-100 (Rhawn, #9002-93-1). Glyburide (200 μM) were pre-incubated with HCECs for 2 h before HKCA (MOI = 20:1) treatment for 24 h. The same LDH release assay method was used to detected the changes of LDH.

### Reverse Transcription-Quantitative Polymerase Chain Reaction (RT-qPCR)

Total RNA was extracted from the corneas of mice and HCECs by TRIzol reagent (Invitrogen, #15596018) according to the manufacturer's instructions. After being synthesized with the iScript cDNA synthesis kit (Bio–Rad, #1708891), cDNA was amplified via SYBR Green qPCR Master Mix (Bio–Rad, #1725121) on a real-time PCR instrument (Light Cycler 480 II, Roche, Switzerland). The relative mRNA expression was analyzed based on the comparative threshold cycle (2^−ΔΔCT^) method and standardized to GAPDH. The primer sequences used in this study are shown in Table 1.

**Table 1:** Primer sequences used for RT-qPCR.

**Gene**	**Forward Primer (5^′^– 3^′^)**	**Reverse Primer (5^′^– 3^′^)**
NLRP3 (mouse)	TGTGAGAAGCAGGTTCTACTCT	GGATGCTCCTTGACCAGTTGG
ASC (mouse)	CAGCACAGGCAAGCACTCA	GGTGGTCTCTGCACGAACT
Caspase-1(mouse)	GGGACCCTCAAGTTTTGCC	GACGTGTACGAGTGGTTGTATT
IL-1β (mouse)	CGCAGCAGCACATCAACAAGAGC	TGTCCTCATCCTGGAAGGTCCACG
IL-18 (mouse)	GACTCTTGCGTCAACTTCAAGG	CAGGCTGTCTTTTGTCAACGA
GSDMD (mouse)	GCGATCTCATTCCGGTGGACAG	TTCCCATCGACGACATCAGAGAC
GADPH (mouse)	AGGTCATCCCAGAGCTGAACG	CACCCTGTTGCTGTAGCCGTAT
NLRP3 (human)	CGTGAGTCCCATTAAGATGGAGT	CCCGACAGTGGATATAGAACAGA
ASC (human)	TGGATGCTCTGTACGGGAAG	CCAGGCTGGTGTGAAACTGAA
Caspase-1 (human)	TTTCCGCAAGGTTCGATTTTCA	GGCATCTGCGCTCTACCATC
IL-1β (human)	AGCTACGAATCTCCGACCAC	CGTTATCCCATGTGTCGAAGAA
IL-18 (human)	ATCGCTTCCTCTCGCAACAA	CTTCTACTGGTTCAGCAGCCATCT
GSDMD (human)	TCACAACCTTGGGGCATCAG	TCCTTCCTGCAAGCTGGTTC
GADPH (human)	TGAACGGGAAGCTCACTGG	TCCACCACCCTGTTGCTGTA

### Western Blot Analysis

Total proteins were extracted from mice corneas or HCECs using a cell lysis buffer (Solabao, #R0010) and measured by the BCA protein assay kit (Thermo Scientific, #23227). Equivalent protein samples were separated by 4-20% sodium dodecyl sulfate-polyacrylamide gel electrophoresis (SDS-PAGE) (Bio-Rad, USA), and then transferred to PVDF membranes (Millipore, USA). The membranes were blocked with 5% skimmed milk diluted in TBST for 1 h and then incubated with primary antibodies at 4°C overnight. Primary antibodies targeting NLRP3 (1:500, ABcam, #ab263899), CASP1 (1:200, ABclonal, #A0964), ASC (1:200, Santa Cruz Biotechnology, #sc-514414), IL-1β (1:200, CST, #63124 or #83186), IL-18 (1:200, ABclonal, #A16737), GSDMD (1:500, Novus Biologicals, #NBP2-33422), and β-actin (1:1000, ABcam, #ab8226) were used. Then, the membranes were incubated with HRP-conjugated secondary antibodies (1:2000, CST, #7074 or #7076) for 1 h at room temperature. The immunoblots were detected with enhanced chemiluminescence (ECL, Thermo Scientific, #32106) reagents and recorded by Image Lab software (Bio–Rad, USA).

### Immunocytochemistry and Immunofluorescence Staining

Mouse corneal sections and HCECs on 6-chamber slides were fixed with 4% paraformaldehyde (Solarbio, #P1110) and permeabilized with 0.2% Triton X-100 (Sigma–Aldrich, #9036-19-5) at room temperature for 10 min. The samples were then incubated with NLRP3 (1:50, Novus Biologicals, #NBP2-12446SS), ASC (1:50, Santa Cruz Biotechnology, #sc-514414), CASP1 (1:200, ABclonal, #A0964), GSDMD (1:100, ABclonal, #A18281) and Ly-6G antibodies (1:200, Servicebio, #GB11229) at 4°C overnight, followed by incubation at room temperature with secondary antibodies (1:300, Servicebio, #GB21303) for 1 h. For TdT-mediated dUTP nick end labeling (TUNEL) staining, the sections were incubated with TUNEL dye (Beyotime, #1086) for 30 min under shade. Nuclei were labeled with DAPI for 10 min. Finally, the samples were observed and photographed with a Leica TCS SP5 confocal microscope (Leica, Germany). For further detection the pyroptotic cells in corneal tissues, double-immunofluorescence staining of active CASP1 and TUNEL was performed on corneal sections. Active CASP1^+^/TUNEL^+^ cells were determined as pyroptotic cells (24).

### Statistical Analysis

Statistical analysis was performed using GraphPad Prism 6.0 software (GraphPad Software, Inc. United States). All experiments were performed at least three times. The data are presented as the mean ±standard error of the mean (SEM) and were analyzed by Student's *t*-test or one-way ANOVA with the LSD *post hoc* test. *P* < 0.05 was considered to indicate statistical significance.

## Results

### NLRP3 Expression Is Markedly Upregulated in Mouse Corneas of *C. albicans* Keratitis

**Figure 1:**
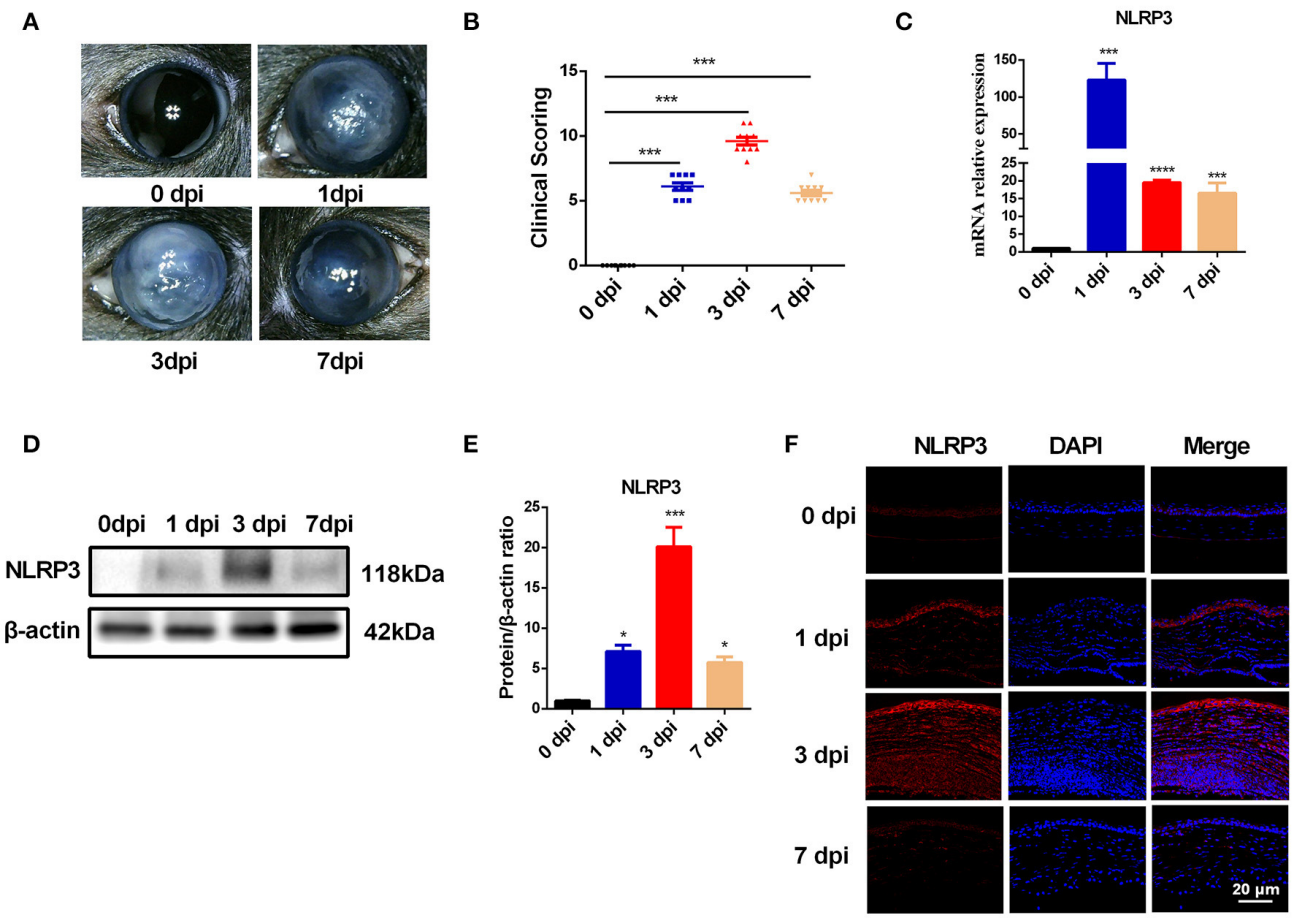
The expression of NLRP3 is upregulated in mouse corneas of *C*. albicans keratitis. C57BL/6 mouse corneas were inoculated with 10^6^ CFU of *C. albicans* or with sterile PBS and photographed daily after the inoculation. **(A)** The photographs of mouse *C. albicans* keratitis were taken by a slit lamp on 0 day (control), 1 day, 3 days, and 7 days post infection (dpi). **(B)** The clinical score of mouse *C. albicans* keratitis at different times during *C. albicans* infection (*n* = 10). RT-qPCR analysis **(C)**, western blot **(D,E)** and immunofluorescence staining **(F)** showing the relative expression of NLRP3 in *C. albicans*-infected mouse corneas at mRNA (*n* = 3) and protein levels (*n* = 3), respectively. Scale bar = 20 μm; magnification 400×. All values are presented as mean ± SEM. *p < 0.05; ***p < 0.001; ****p < 0.0001 vs. control group.

To explore the molecular events initiated by *C. albicans*-infected corneas, an experimental murine model of *C. albicans* keratitis was successfully created. Consistent with our previous observations (22), all infected eyes developed obvious clinical signs of fungal keratitis (Figure 1A), and corneal inflammation started at 1 dpi (mean score 6.1 ± 0.9), peaked at 3 dpi (9.6 ± 1.0), and then was mitigated at 7 dpi (5.6 ± 0.7) (Figure 1B). To determine whether NLRP3 inflammasome activation and pyroptosis are involved in *C. albicans* keratitis, we first verified NLRP3 expression in *C. albicans*-infected corneas. As shown in Figure 1C, the mRNA level of NLRP3 was significantly upregulated 122-fold at 1 dpi, 19-fold at 3 dpi, and 16-fold at 7 dpi compared to the mock controls. Western blot analysis also indicated significantly upregulated expression of NLRP3 protein in the cornea at different times during *C. albicans* infection (Figures 1D,E). Moreover, immunofluorescence staining further revealed that the elevated levels of NLRP3 protein were located in the corneal epithelium and stromal cells in the *C. albicans* keratitis model (Figure 1F). Overall, these results indicated that NLRP3 is upregulated in mouse corneas following *C. albicans* infection, and prompted us to investigate the role of NLRP3 inflammasome in *C. albicans* keratitis.

### *C. albicans* Infection Triggers Pyroptosis in Mouse Corneas

**Figure 2:**
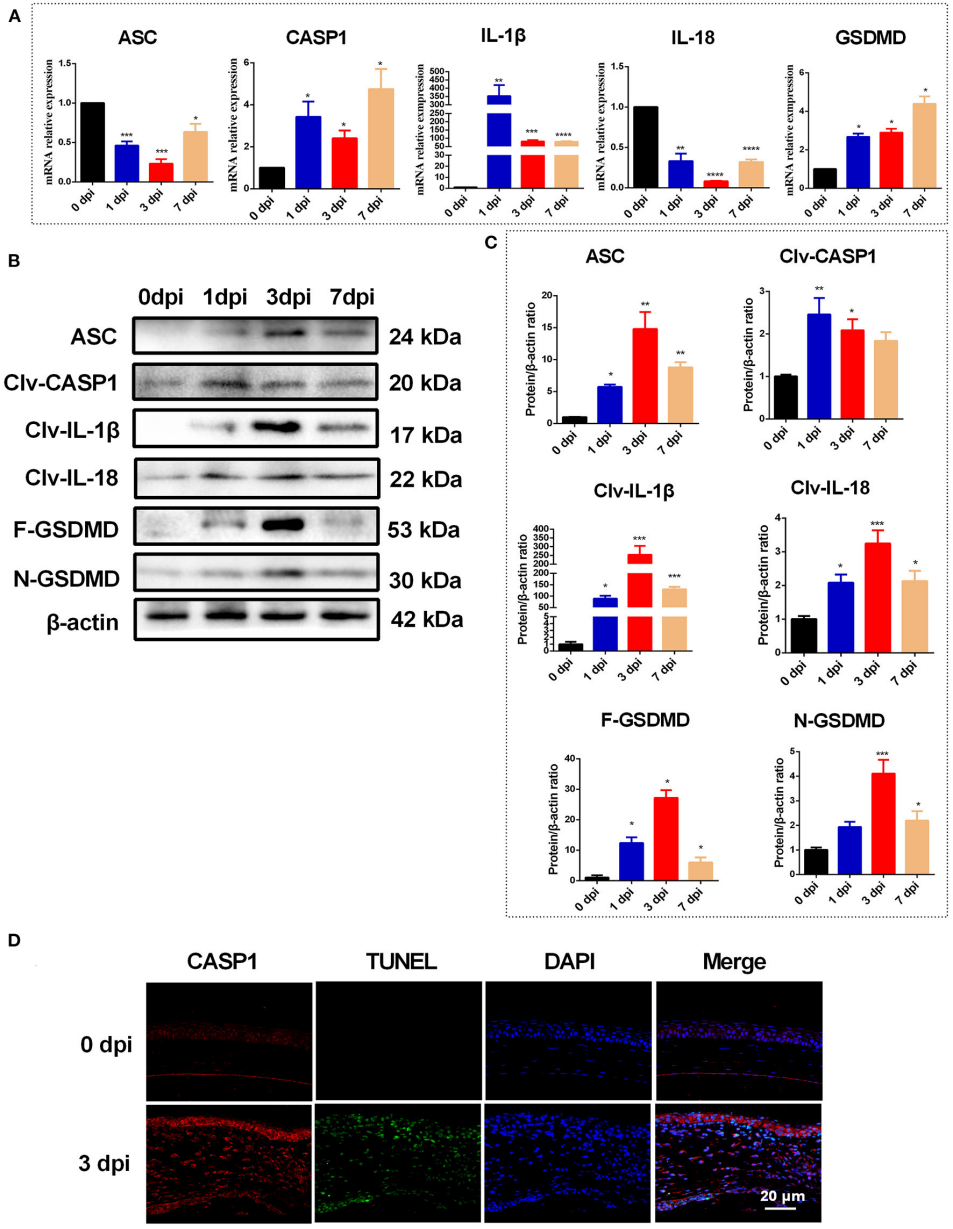
Pyroptosis is occurred in mouse corneas of *C. albicans* keratitis. **(A)** RT-qPCR analysis of the mRNA levels of pyroptosis-associated genes (ASC/CASP1/GSDMD/IL-1β/IL-18) in mouse corneas at 0 (control), 1, 3, and 7 dpi (*n* = 3). **(B,C)** Western blot detecting pyroptosis-related proteins of ASC, cleaved CASP1, cleaved IL-1β, cleaved IL-18, F-GSDMD and N-GSDMD in mouse corneas at 0 (control), 1, 3, and 7 dpi (*n* = 3). **(D)** Double-immunofluorescence staining of CASP1 and TUNEL in *C. albicans* infected-corneas compared with mock-infected controls (*n* = 3; Scale bar = 20 μm; magnification 400×). CASP1: caspase-1; Clv-CASP1:cleaved CASP1; Clv-IL-1β:cleaved IL-1β; Clv-IL-18:cleaved IL-18; F-GSDMD: p53 form of GSDMD; N-GSDMD: cleaved p30 form of GSDMD. All values are presented as mean ± SEM. *p < 0.05; **p < 0.01; ***p < 0.001; ****p < 0.0001 vs. control group.

During fungal infection, the activation of the NLRP3-ASC-CASP1 inflammasome leads to cleavage of pro-CASP1, which then processes the proinflammatory cytokines IL-1β and IL-18 to their bioactive forms and cleaves GSDMD to trigger pyroptosis (9). Thus, the expression of ASC, CASP1, IL-1β, IL-18, and GSDMD is expected to be significantly increased during pyroptosis. As shown in Figure 2A. *C. albicans* infection significantly increased the mRNA levels of CASP1, GSDMD and IL-1β in mouse corneas. However, compared with the control group, the mRNA levels of ASC and IL-18 were significantly reduced in *C. albicans*-infected mouse corneas (Figure 2A). Western blot analysis showed that the protein levels of ASC, cleaved CASP1, N-GSDMD, cleaved IL-1β and cleaved IL-18 were all significantly elevated in *C. albicans* keratitis (Figures 2B,C). Interestingly, the increased expression of ASC and IL-18 in *C. albicans* keratitis was only observed at the protein level but not the mRNA level, which may be attributed to the posttranslational regulation of these genes, but this conclusion needs further confirmation. To further confirm the presence of pyroptosis in *C. albicans*-infected mouse corneas, we performed double-immunofluorescence staining. Active CASP1 and TUNEL double positive cells were designated as pyroptotic cells (25). As shown in Figure 2D, pyroptotic cell death was obviously observed in *C. albicans*-infected corneas, in contrast, no pyroptotic cell was observed in control group. These findings suggested that pyroptosis occurred in *C. albicans* keratitis.

### NLRP3 Knockdown Ameliorates Corneal Damage and Inflammation in Mouse Corneas During *C. albicans* Infection

**Figure 3:**
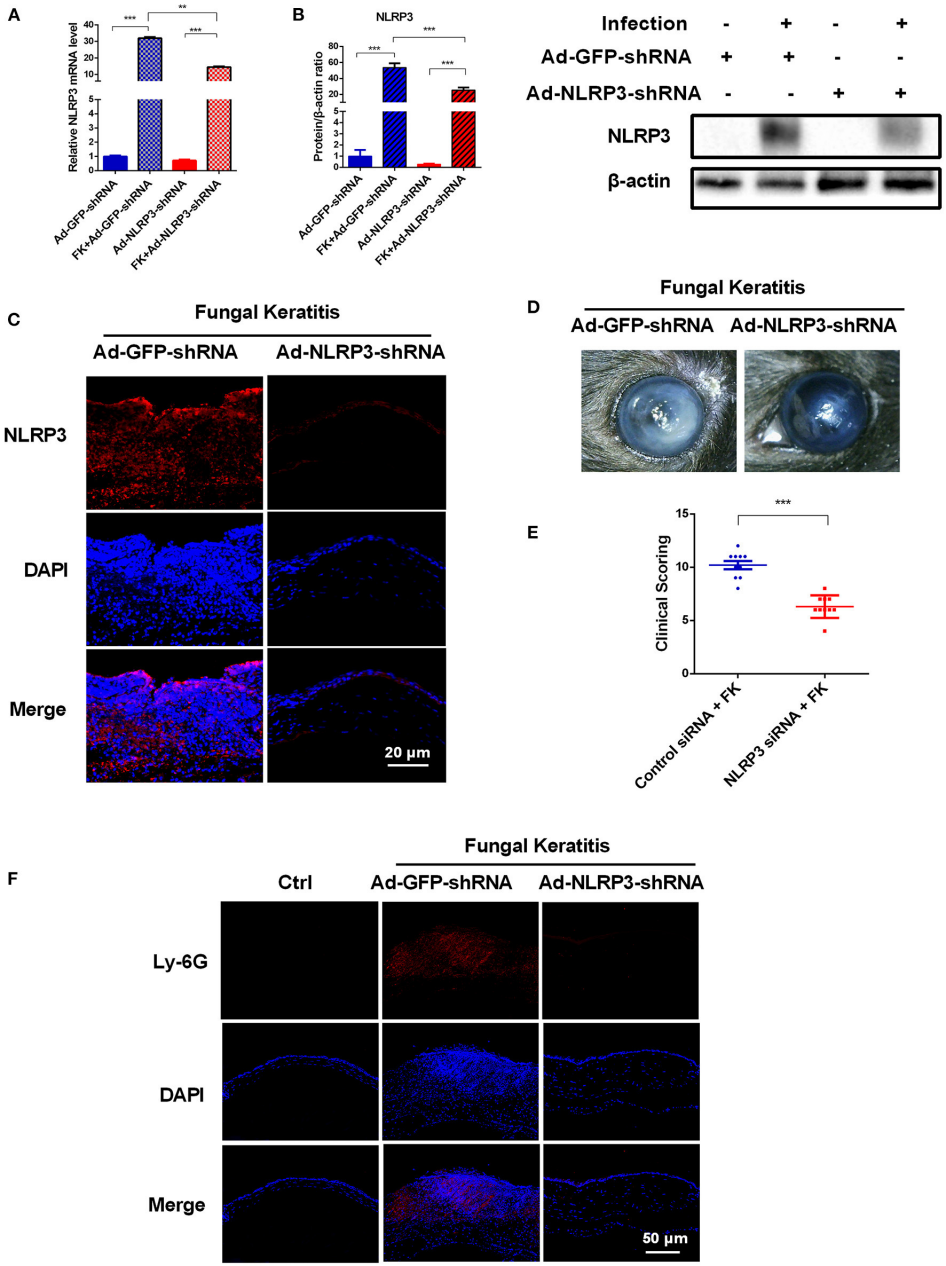
NLRP3 knockdown decreases corneal inflammation and suppressed neutrophil infiltration in mouse *C. albicans* keratitis. The C57BL/6 mice were subconjunctivally injected with 6 μL (4 × 10^8^ PFU) of the Ad-GFP-shRNA or Ad-NLRP3-shRNA suspension 3 days before inoculation with 1 × 10^6^ CFU *C. albicans* or with 5μL sterile PBS after the corneas were scratched. The mouse corneas or eyeballs were collected at 3 dpi and subjected for further detection. RT-qPCR analysis **(A)**, western blot **(B)** and immunofluorescence staining **(C)** were used to verify the gene knockdown efficiency of Ad-NLRP3-shRNA (*n* = 3). Scale bar = 20 μm; magnification 400×. **(D)** Micrographs of Ad-GFP-shRNA and Ad-NLRP3-shRNA-pretreated mouse corneas were photographed at 3dpi. **(E)** Clinical score of the infected corneas pretreated with Ad-GFP-shRNA and Ad-NLRP3-shRNA (*n* = 10). **(F)** Immunofluorescence staining was performed to assess the levels of neutrophils recruitment in mouse corneas after Ad-GFP-shRNA and Ad-NLRP3-shRNA pretreatment (*n* =3). Scale bar = 50 μm; magnification 200×. FK: fungal keratitis. All values are presented as mean ± SEM. **p < 0.01; ***p < 0.001.

To further explore the contributions of the NLRP3 inflammasome during *C. albicans* infection, adenovirus-mediated shRNA was used to knockdown NLRP3 expression in mouse corneas. As shown in Figures 3A–C, NLRP3 mRNA and protein levels were significantly reduced after selective NLRP3 knockdown. Importantly, compared with that in the Ad-GFP-shRNA group, fungal keratitis with Ad-NLRP3-shRNA treatment showed much less corneal inflammation and clinical scores (Figures 3D,E). Moreover, Ly-6G immunofluorescence staining was used to measure neutrophil infiltration after NLRP3 knockdown at 3 d after infection. As shown in Figure 3F, there was almost no significant positive staining in Ad-NLRP3-shRNA group compared with the Ad-GFP-shRNA group. These results indicated that NLRP3 inflammasome activation is involved in the progression of *C. albicans* keratitis.

### The NLRP3 Inflammasome Is a Vital Mediator of Pyroptosis in *C. albicans* Keratitis

**Figure 4:**
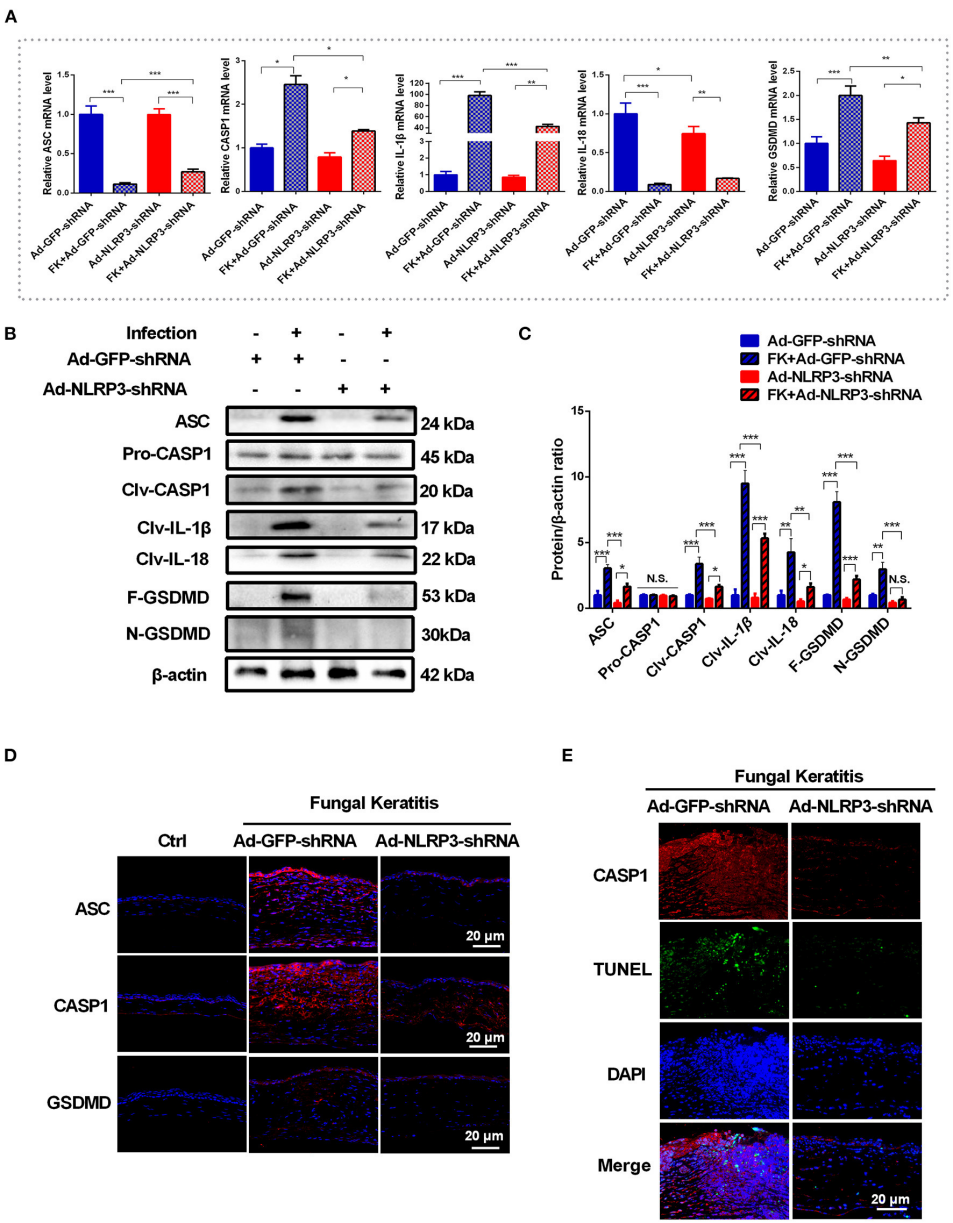
NLRP3 knockdown attenuates the pyroptosis in mouse *C. albicans* keratitis. **(A–C)** RT-qPCR analysis and western blot showing the mRNA and protein levels of pyroptosis-related molecules in *C. albicans*-infected corneas pretreated with Ad-GFP-shRNA and Ad-NLRP3- shRNA compared with mock controls (*n* = 3). **(D)** Immunofluorescence staining of ASC, CASP1 and GSDMD in *C. albicans*-infected corneas pretreated with Ad-GFP-shRNA and Ad-NLRP3- shRNA compared with mock controls (*n* = 3). **(E)** Double-immunofluorescence staining of CASP1 and TUNEL in the mice cornea of Ad-NLRP3-shRNA group compared with the Ad-GFP-shRNA group (*n* = 3). Scale bar = 20 μm; magnification 400×. FK: fungal keratitis. CASP1: caspase-1; Clv-CASP1:cleaved CASP1; Clv-IL-1β:cleaved IL-1β; Clv-IL-18:cleaved IL-18; F-GSDMD: p53 form of GSDMD; N-GSDMD: cleaved p30 form of GSDMD. All values are presented as mean ± SEM. *p < 0.05; **p < 0.01; ***p < 0.001.

NLRP3 inflammasome activation is essential for fungal infection*-*induced pyroptosis in macrophages (9, 11). To further identify whether pyroptosis is initiated by activation of the NLRP3 inflammasome in *C. albicans* keratitis, the expression of pyroptosis-associated markers (ASC/CASP1/GSDMD/IL-1β/ IL-18) was determined in mouse corneas after selective NLRP3 knockdown. As shown in Figure 4A, after *C. albicans* infection, NLRP3 knockdown significantly suppressed the increase in CASP1, GSDMD and IL-1β in mouse corneas at the mRNA level but not that of ASC or IL-18. Moreover, western blot analysis revealed dramatically decreased levels of pyroptosis-associated proteins ASC, cleaved CASP1, GSDMD, cleaved GSDMD, cleaved IL-1β and cleaved IL-18 in the Ad-NLRP3-shRNA group compared with the Ad-GFP-shRNA group during fungal infection (Figures 4B,C). However, the protein level of pro-CASP1 was not affected by NLRP3 knockdown (Figures 4B,C). Immunofluorescence staining further verified that the protein levels of ASC, CASP1 and GSDMD were markedly increased in the corneal epithelium and stromal cells of the Ad-GFP-shRNA group during *C. albicans* infection, while this increase was significantly suppressed by NLRP3 knockdown (Figure 4D). Meanwhile, NLRP3 knockdown attenuated pyroptosis in *C. albicans*-infected mice cornea according to double-immunofluorescence staining of active CASP1 and TUNEL (Figure 4E). These findings suggested that *C. albicans* infection-induced pyroptosis in mouse corneas was dependent on NLRP3 inflammasome activation.

### NLRP3 Inflammasome and Pyroptosis Are Activated in HCECs Exposed to HKCA

**Figure 5:**
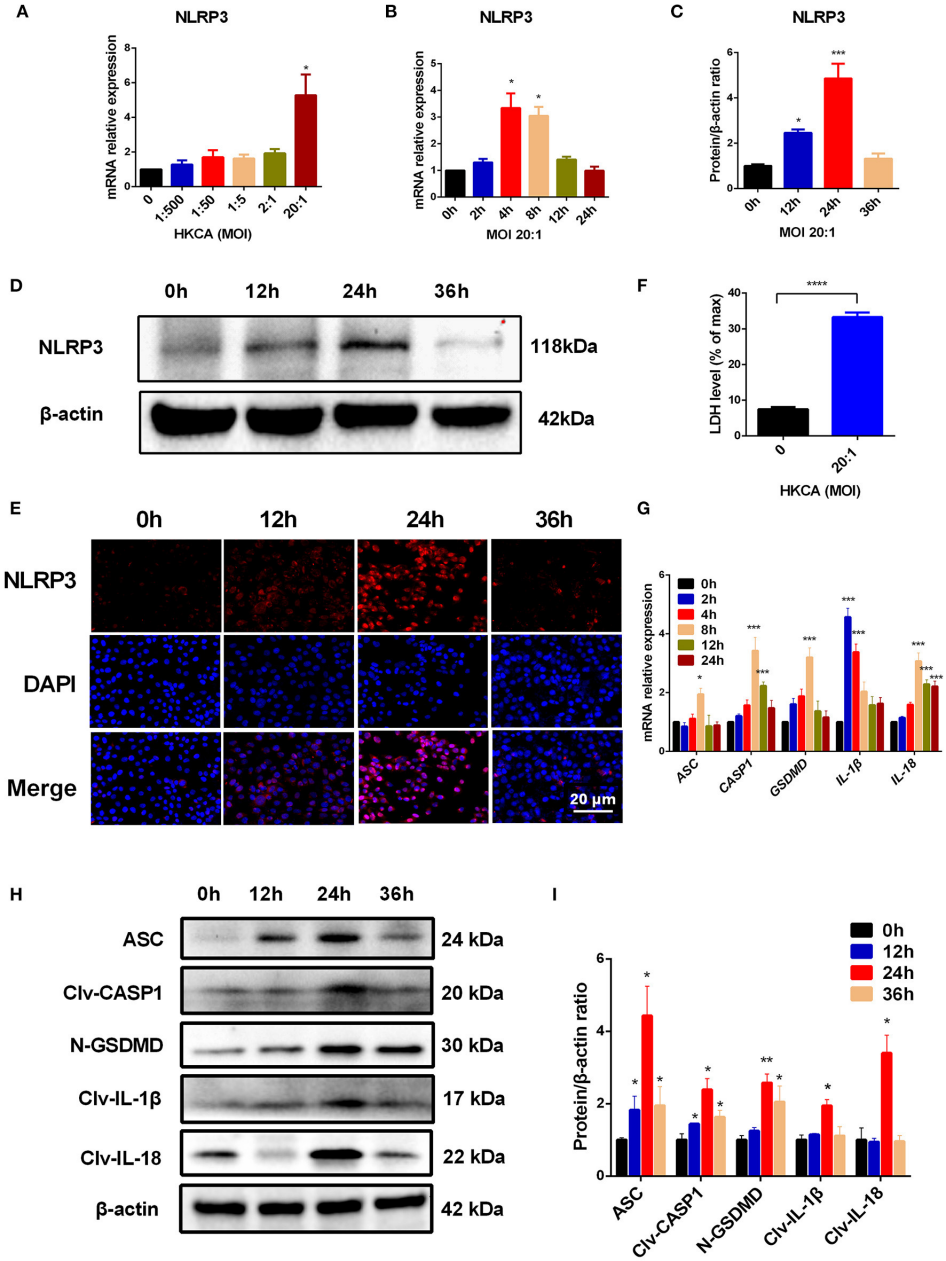
Heat-killed *C. albicans* (HKCA) activates NLRP3 inflammasome and induces pyroptosis in human corneal epithelial cells (HCECs). **(A)** The mRNA expression of NLRP3 in HCECs challenged with HKCA at an MOI of 1:500, 1:50, 1:5, 2:1, or 20:1 respectively for 4 hours was evaluated by RT-qPCR (*n* = 5). **(B–D)** The mRNA and protein expression of NLRP3 in HCECs exposed to HKCA (MOI = 20) for 0 (control), 2, 4, 8, 12, or 24 h (*n* = 3). **(E)** NLRP3 fluorescence intensity was evaluated using immunofluorescent staining for different times (12–36 h). (*n* = 3; Scale bar = 20 μm; magnification 400×). **(F)** Lactate dehydrogenase (LDH) of HCECs treated with HKCA (MOI = 20) for 24 h (*n* = 6). **(G)** The mRNA levels of ASC, CASP1, IL-1β, IL-18 and GSDMD in HCECs exposed to HKCA (MOI = 20) for different times (*n* = 3). **(H,I)** The protein expression of pyroptosis-related proteins (ASC, cleaved CASP1, N-GSDMD, cleaved IL-1β and cleaved IL-18) was examined by western blot (*n* = 3). CASP1: caspase-1; Clv-CASP1: cleaved CASP1; Clv-IL-1β: cleaved IL-1β; Clv-IL-18: cleaved IL-18; N-GSDMD: cleaved p30 form of GSDMD. All values are presented as mean ± SEM. *p < 0.05; **p < 0.01; ***p < 0.001; ****p < 0.0001 vs. control group.

Having established the crucial role of NLRP3 inflammasome-mediated pyroptosis in the pathogenesis of *C. albicans*-infected mouse corneas, we sought to further assess whether this pathway was important in corneal epithelial cells *in vitro*. We first measured the expression of NLRP3 and pyroptosis-related markers (ASC/CASP1/GSDMD/IL-1β/IL-18) in HCECs after incubation with HKCA. As shown in Figure 5A, exposure of HCECs to HKCA at an MOI of 1:500, 1:50, 1:5, 2:1, or 20:1 respectively for 4 h resulted in a dose-dependent increase in NLRP3 at the mRNA level. HKCA at an MOI of 20:1 markedly stimulated NLRP3 transcription up to 5.3-fold compared with that in controls (*p* < 0.05). Therefore, HKCA at an MOI of 20:1 was used to stimulate HCECs in subsequent experiments. We further found that the mRNA expression of NLRP3 fluctuated according to the HKCA stimulation times (Figure 5B). Similarly, the western blot results and immunofluorescence staining showed that the protein expression of NLRP3 was significantly increased at 12 h, peaked at 24 h and decreased at 36 h in HKCA-challenged HCECs (Figures 5C–E). During pyroptosis, N-GSDMD forms pores in the plasma membrane, leading to the release of cellular contents from dead cells, which can be measured by the LDH release assay. The results of the LDH release assay indicated that cell death in HKCA-induced HCECs was obviously higher than that in control group (Figure 5F). Furthermore, we observed that both the mRNA and protein levels of pyroptosis-related markers were upregulated after HKCA (MOI = 20) treatment (Figures 5G–I). Thus, HKCA exposure activates the NLRP3 inflammasome and pyroptosis in HCECs.

### Glyburide Inhibits HKCA-Induced HCEC Pyroptosis Triggered by the NLRP3 Inflammasome

**Figure 6:**
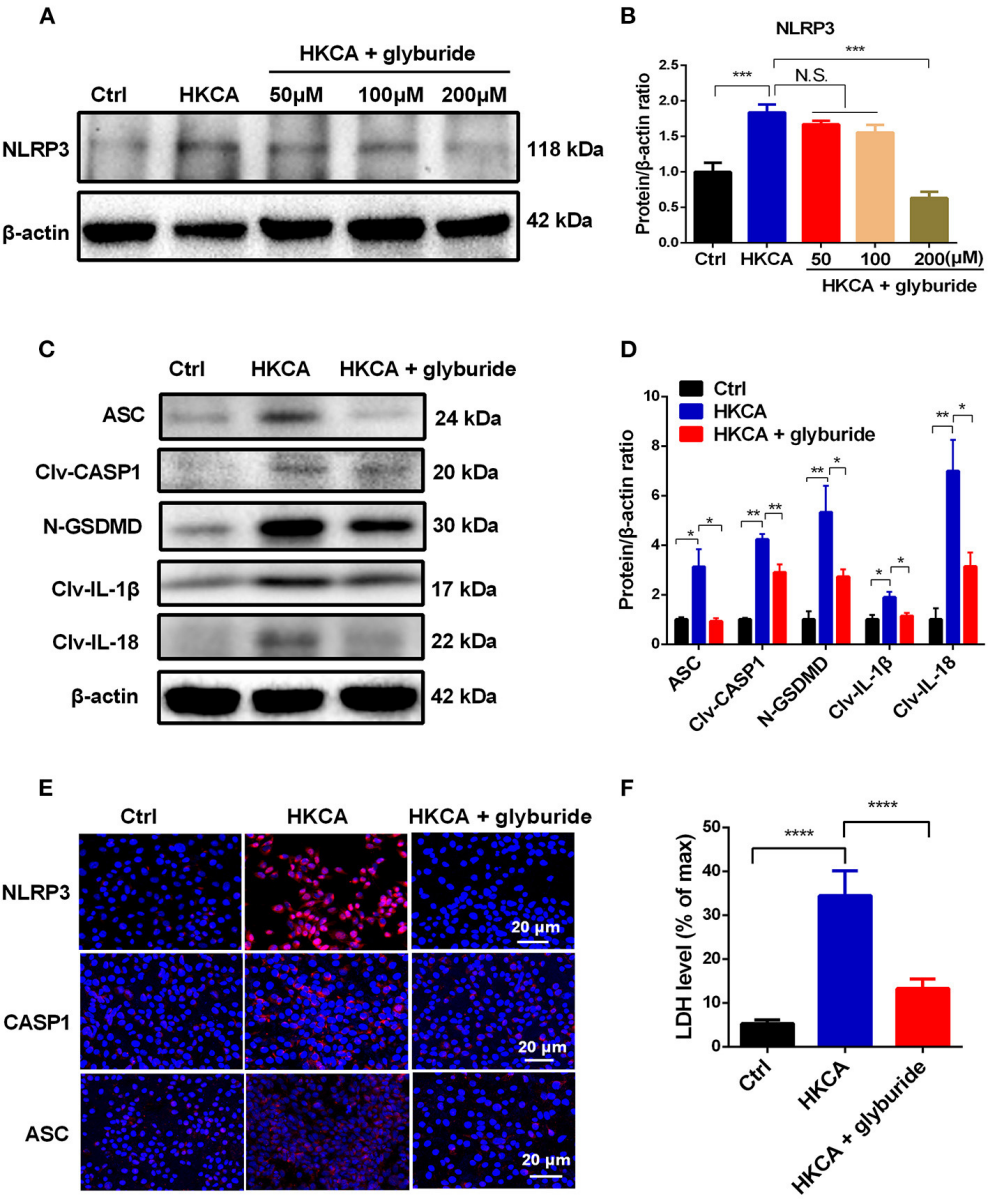
Glyburide attenuates NLRP3 inflammasome-mediated pyroptosis in HCECs infected with HKCA. HCECs were pretreated with potassium (K^+^) channel inhibitor (glyburide) for 2 h, and then were incubated with HKCA (MOI = 20) for 24 h. **(A,B)** Western blot showing the protein levels of NLRP3 in HCECs treated with various concentrations of glyburide (50, 100 and 200 μM) (*n* = 3). **(C,D)** Glyburide treatment (200 μM) suppressed the levels of pyroptosis-related proteins (ASC, cleaved CASP1, N-GSDMD, cleaved IL-1β and cleaved IL-18) in HCECs challenged with HKCA at 20:1 for 24 h (*n* = 3). **(E)** Immunofluorescence analysis of NLRP3, CASP1 and ASC in HCECs pretreated with or without glyburide (200 μM) for 24 h (*n* = 3). Scale bar = 20 μm; magnification 400×. **(F)** LDH release of HCECs treated with glyburide (200 μM) (*n* = 6). CASP1: caspase-1; Clv-CASP1: cleaved CASP1; Clv-IL-1β: cleaved IL-1β; Clv-IL-18: cleaved IL-18; N-GSDMD: cleaved p30 form of GSDMD. All values are presented as mean ± SEM. N.S. P>0.05; *p < 0.05; **p < 0.01; ***p < 0.001; ****p < 0.0001.

Glyburide was used as an ATP-sensitive K^+^ (K_ATP_) channel antagonist to prevent NLRP3 inflammasome activation and microbial ligand-induced IL-1β secretion (26). To further address whether HKCA-induced pyroptosis in HCECs was initiated by NLRP3 inflammasome activation and to determine the effect of K^+^ efflux on NLRP3 inflammasome activation, we conducted glyburide inhibitory experiments. Our results suggest that 200 μM glyburide significantly reversed the HKCA-induced upregulation of NLRP3 and pyroptosis-related proteins compared with those in the untreated group (Figures 6A–D). Moreover, immunofluorescence staining further confirmed that 200 μM glyburide treatment significantly suppressed the elevation in NLRP3, CASP1 and ASC at the protein level induced by HKCA (Figure 6E). To assess whether glyburide also affects pyroptosis, we measured the release of the LDH in HCECs. As shown in Figure 6F, 200 μM glyburide treatment markedly alleviated the increase of cell death in HKCA-induced HCECs. Taken together, these findings provide firm evidence that HKCA can trigger the NLRP3 inflammasome-mediated pyroptosis pathway in HCECs, and this pathway may be related to K^+^ efflux and can be blocked by glyburide.

## Discussion

The present study as the first to provide evidence that the expression of NLRP3 inflammasome and GSDMD was significantly elevated in mouse *C. albicans* keratitis and in HCECs exposed to HKCA. Importantly, knockdown of NLRP3 decreased pyroptosis and successfully reversed corneal inflammation in *C. albicans* keratitis. Our data also indicated that HKCA-induced HCECs pyroptosis was initiated by NLRP3 inflammasomes, and this activation is associated with K^+^ efflux because treatment with the K^+^ channels inhibitor glyburide resulted in a significant decrease in NLRP3/CASP1/GSDMD activation, as well as IL-1β/IL-18 secretion and LDH release. Taken together, these findings suggested that *C. albicans* infection could induce NLRP3 inflammasome-mediated pyroptosis in mouse corneas and HCECs. Additionly, NLRP3 inflammasome-mediated pyroptosis during *C. albicans* keratitis is closely related to the inflammatory injury of cornea. Thus, blocking the activation of the NLRP3 inflammasome-mediated pyroptosis pathway may provide a new therapeutic strategy for fungal keratitis.

Although the pathogenesis of fungal infectious diseases is not fully understood, inflammasome activation and pyroptosis have been identified as the key mechanisms for host defense during fungal infection (9, 14). Among the inflammasomes, the NLRP3 inflammasome is the most well-studied inflammasome related to fungal infection. During fungal infection, the NLRP3 inflammasome formation in immune cells leads to the activation of CASP1, which then cleaves the proinflammatory cytokines IL-1β and IL-18 to their bioactive forms and induces pyroptosis (9). Currently, several studies have demonstrated that the NLRP3 inflammasome is involved in the pathogenesis of keratitis due to infection with *A. fumigatus or Fusarium* (15–17). Moreover, it was reported that the expression of GSDMD, a pyroptosis executor, was significantly increased in human and mouse *A. fumigatus* keratitis, as well as in HCECs infected with *A. fumigatus*, suggesting that corneal epithelial cells are able to respond to fungal infection via pyroptosis (20). However, there is no evidence showing that NLRP3 inflammasome activation and pyroptosis are observed in the cornea in response to *C. albicans* infection. In the present study, we confirmed the presence of the NLRP3 inflammasome activation and cell pyroptosis in mouse *C. albicans* keratitis. Our results indicated that multiple proteins that were associated with inflammasome and pyroptosis (NLRP3, ASC, CAP1, GSDMD, IL-1β and IL-18) were highly expressed in *C. albicans* keratitis. In addition, immunofluorescence staining showed upregulated expression of NLRP3, ASC, CASP1 and GSDMD in the corneal epithelium and stromal cells of infected mouse corneas. Pyroptosis is an inflammasome and inflammatory CASP (mainly CASP1) mediated programmed cell death (9, 27). In addition, increasing evidences have shown that the DNA fragmentation during pyroptosis can be labeled by TUNEL-staining (28, 29). Therefore, the active CASP1 and TUNEL double-immunofluorescence staining was widely used to detect pyroptosis (24, 25, 30). We found that pyroptotic cells was obviously observed in *C. albicans*-infected mouse corneas according to CASP1 and TUNEL double-immunofluorescence staining. These results revealed that the NLRP3 inflammasome was activated in the cornea to mediate pyroptosis during *C. albicans* infection.

When the NLRP3 inflammasome is activated, a large amount of the proinflammatory cytokines IL-1β and IL-18 are released through GSDMD pores, which may trigger and aggravate corneal inflammation and result in enhanced ocular injury in *C. albicans* keratitis. Moreover, GSDMD is thought to promote the secretion of the cytokines IL-1β and IL-18 (31). Additionally, neutrophils, which account for 95% of the cellular infiltrate in infectious keratitis (17), are considered to be crucial for host defense against fungal pathogens but may also lead to tissue damage, which results in corneal opacity (32). It has been suggested that NLRP3 inflammasome activation and IL-1β secretion are needed to drive the recruitment of neutrophils to the site of inflammation (33, 34). Liang et al. found that tacrolimus combined with natamycin obviously mitigated corneal disorders by inhibiting NLRP3 inflammasome activation in mouse *A. fumigatus* or *Fusarium* keratitis (15). Another recent study on mouse fungal keratitis demonstrated that inhibiting GSDMD expression with GSDMD siRNA could improve the outcome of keratitis, which was characterized by decreased corneal inflammation, suppressed neutrophil and macrophage infiltration and reduced production of IL-1β (20). Broadly consistent with their results, we found that knockdown of NLRP3 markedly reduced pyroptosis and the subsequent production of IL-1β and IL-18, suggesting that *C. albicans*-induced pyroptosis in mouse corneas occurs through NLRP3 inflammasome activation. Importantly, our data provide the first evidence that NLRP3 knockdown also greatly alleviates the severity of corneal infection and reduces the recruitment of neutrophils to the corneal stroma in *C. albicans* keratitis, indicating that the NLRP3 inflammasome not only participates in but also plays a proinflammatory role in *C. albicans* keratitis by triggering pyroptosis in the cornea. Hence, tightly regulating the NLRP3 inflammasome-mediated pyroptosis pathway would suggest new intervention strategies to inhibit corneal inflammation and ameliorate blinding fungal keratitis.

It has been demonstrated that both NF-κB and Dectin-1-dependent signaling are the key pathway involved in fungus-induced NLRP3 priming (14). In our previous study, we found that the innate immune response to HKCA in HCECs is initiated through Dectin-1/ NF-κB signaling (35). Furthermore, Ganesan et al. found that Dectin-1 is required for HKCA-induced cell death in mouse dendritic cells (13). HKCA contains β-glucan, the major cell wall component of fungi, which has been confirmed to activate the NLRP3 inflammasome and drive IL-1β production in both immune and epithelial cells, in line with *C. albicans* infection (13, 35, 36). Therefore, we speculate that HKCA may also be involved in stimulating the activation of NLRP3 inflammasome and pyroptosis. Consistent with our results in mice, the *in vitro* study showed that the NLRP3 inflammasome was activated and pyroptosis was induced in HCECs infected with HKCA. During pyroptosis, a great number of pores are formed on the cell membrane, resulting in the loss of cell membrane integrity, which induces cell lysis and the release of intracellular contents (9), including LDH. We found that LDH levels were significantly increased in HCECs infected with HKCA, further suggesting that *C. albicans* infection induces pyroptotic cell death in the corneal epithelium. To date, the majority of studies on *C. albicans*, the NLRP3 inflammasome and pyroptosis have focused on innate immune cells, such as macrophages and dendritic cells (9, 13). Our research suggests that NLRP3 inflammasomes and pyroptosis are also involved in corneal epithelial cells in response to *C. albicans* infection.

Although the exact mechanism of NLRP3 inflammasome activation has not been sufficiently elucidated, emerging evidence suggests that ROS production and K^+^ efflux are required by almost all fungi to activate the NLRP3 inflammasome. (14). Our previous research has confirmed the important role of ROS in HCECs exposed to *C. albicans* (22), and we here focused on K^+^ efflux. Glyburide is an ATP-sensitive K^+^ (K_ATP_) channel antagonist and potent NLRP3 inhibitor (26, 37). Our previous study showed that glyburide was able to reduce alkali burn-induced ocular surface injury by suppressing the expression of NLRP3 and the release of IL-1β and IL-18 in alkali-injured mouse corneas *in vivo* (38). Cai et al. reported that glyburide significantly inhibited high glucose plus LPS-induced elevation of NLRP3 and CASP1 proteins in peritoneal macrophages *in vitro* (37). Moreover, it has been proven that glyburide markedly attenuated NLRP3 inflammasome-mediated pyroptosis in acute lung injury (39). Consistent with previous studies, our findings showed that treatment with glyburide efficiently suppressed NLRP3 inflammasome activation and pyroptosis in HCECs challenged with HKCA, suggesting that HKCA-induced HCECs pyroptosis through the NLRP3 inflammasome pathway, by which K^+^ efflux may contribute to the activation of the NLRP3 inflammasome. However, the mechanism by which glyburide inhibits NLRP3 inflammasomes and suppresses IL-1β secretion has been suggested to be an is an intriguing question (37). Gross et al. found that *C. albicans* inflammasome activation and IL-1β production were inhibited via blocking K^+^ efflux by using glyburide (40). Lamkanfi et al. demonstrated that the ability of glyburide to inhibit NLRP3 activation is independent of its inhibitory effect on K_ATP_ channels and speculated that glyburide acts the upstream of NLRP3 and the downstream of P2X7 receptor (26). However, the exact molecular target of glyburide inhibiting NLRP3 inflammasome has yet to be elucidated (37).

However, this study still has some limitations. In addition to the canonical NLRP3 inflammasome-mediated pyroptosis pathway, the involvement of other inflammasomes or noncanonical pyroptosis pathways in corneal damage in *C. albicans* keratitis remains unclear and needs further investigation. Additionally, the normal cornea contains relatively few resident immune cells, such as macrophages and dendritic cells (32). Besides corneal epithelial cells, NLRP3 is also expressed in macrophages and dendritic cells (11, 13). Although our *in vitro* experiments showed that inhibition of NLRP3 suppressed pyroptosis in HCECs, the deletion of NLRP3 in resident immune cells may also be involved in alleviation of corneal inflammation by treating mice with Ad-NLRP3-shRNA *in vivo*. Future studies will explore the roles of NLRP3-expressing immune cells in the corneal tissue except epithelium on pyroptosis in *C. albicans* keratitis.

In summary, our data demonstrated for the first time that NLRP3 inflammation-mediated pyroptosis plays an important role in mediating the immune response during *C. albicans* keratitis. Therefore, targeting NLRP3 inflammasome-mediated pathway may be a potential target that could be used to prevent or treat *C. albicans* keratitis.

## Data Availability Statement

The original contributions presented in the study are included in the article/supplementary material, further inquiries can be directed to the corresponding author/s.

## Ethics Statement

The animal study was reviewed and approved by Animal Ethics and Welfare Committee of Research Center for Eco-Environmental Sciences, Chinese Academy of Sciences (No. AEWC-RCEES-2021032).

## Author Contributions

XY conceived and designed the study. HL and XF performed the animal and cell experiments and wrote the paper. HL, SL, and QW performed the analyses. QL, XH, WL, and XY conducted the analyses. XY and CL reviewed and edited the manuscript. All authors revision and approval of the manuscript. All authors contributed to the article and approved the submitted version.

## Funding

This work was supported by the National Natural Science Foundation of China (Nos. 81970772, 81870638, 81670817, and 81670816) and the Science and Technology Program of Baoding of Huifang Lian (No. 2141ZF086).

## Conflict of Interest

The authors declare that the research was conducted in the absence of any commercial or financial relationships that could be construed as a potential conflict of interest.

## Publisher's Note

All claims expressed in this article are solely those of the authors and do not necessarily represent those of their affiliated organizations, or those of the publisher, the editors and the reviewers. Any product that may be evaluated in this article, or claim that may be made by its manufacturer, is not guaranteed or endorsed by the publisher.
